# The Local Government Board's Annual Report

**Published:** 1914-01-24

**Authors:** 


### THE ADMINISTRATION OF THE POOR LAW.*

### The Local Government Board's Annual Report.

To those who are only too ready to set aside the Poor Law and all that pertains to it as " costly and horrible " (for so the Poor-Law system was recently described) we commend the study of Part I. of the Forty-second Annual Report of the Local Government Board, 1912-1913.

We recently noted very briefly some of the statistics of the Report. Of the " indoor paupers " receiving relief on January 1, 1913, no fewer than 99,778, or more than 36 per cent. of the total number, were allocated in infirmaries, infirm wards, or hospitals. On November 4, 1911, " nearly one-third of the persons (other than lunatics and casuals) in receipt of Poor-Law relief in England and Wales were under medical treatment or care, and the proportion was considerably higher in London; the proportion of indoor paupers under medical treatment or care was, as might be expected, far higher than that of the outdoor paupers." Of 10,330 paupers under treatment for pulmonary tuberculosis in England and Wales, London, Lancashire, and the West Riding of Yorkshire accounted for more than half; the 2,767 cases of pulmonary tuberculosis represented 7 per cent. of the total number of paupers under medical treatment or care in London. In only too many cases these consumptive patients, the large majority of whom are males, are fathers of families also wholly or in part dependent upon Poor-Law relief.

In spite of the undoubted tendency shown of recent years to mark time in Poor-Law work in view of possible legislation, " progress continues to be made in the provision of suitable accommodation for the sick poor " and of separate accommodation for children. " It is beyond question that sick persons are now apt to resort more freely than formerly to Poor-Law institutions," this tendency having grown concurrently with the excellence of the treatment provided. The fact that the total number of women under Poor-Law medical treatment exceeds that of men is mainly due to the preponderance of women over fifty years of age who were under treatment, especially at their own homes; the majority of the men over fifty years of age are under treatment in institutions. To the large London institutions, however, as to those in other places where rents are high, the aged tend to gravitate, and the proportion of women is large—especially in the case of the bodily and mentally infirm.

Especial interest attaches to the Report in connection with nursing and with the maternity wards. With the steady improvement in the quality of the nursing work in Poor-Law institutions the number of nurses has continued to increase, whilst reliance upon the doubtful assistance of " able-bodied " inmates and others has diminished to the vanishing point. In 1883 the number of nurses employed was 2,385, in 1893 it was 2,994, in 1903 it was 5,674, and in 1913 it reached 7,652. In the district comprising the Union Counties of Durham, Northumberland, and Yorkshire North Riding, whilst the paid nurses in 1903 only numbered one to every 18.76 patients, they had been increased by 1912 to one to every 12.94, a proportion still too low in view of the requirements of modern treatment.

" The qualifications of the nurses have improved." Every year approximately 400 probationers complete a three-years' course of training in the Poor-Law infirmaries of the Metropolis, and nearly 1,600 are there undergoing such training; many provincial schools also give a systematic training. Provision is often made for training in midwifery as an addition to the general nursing training. Mr. C. F. Roundell reports:—" An increasing difficulty is being found in obtaining nurses . . . even where good salaries are paid. There can be little doubt that the conditions of a nurse's life in a country workhouse need improvement in order to attract and, what is more important, if possible, to retain suitable persons. The life is hard, and in many ways monotonous, and nurses have a right to expect comfortable quarters and properly cooked and properly served food." Mr. W. P. Elias, who also refers to " the shortage in the supply of trained nurses now apparently existing," adds much of great interest upon the nursing question. He quotes a paper by Dr. S. S. Whillis in which " the creation of a joint examination board by the Poor-Law training institutions on the north-east coast " is suggested. A similar proposal has been made in London. It would seem, however, to be unwise to incur the risk, by the initiation of Poor-Law nurses' examining bodies, of appearing to separate such nurses as a class apart. Should central examinations, such as have been arranged by the Central Midwives Board, be held for nurses after general training, to them Poor-Law nurses should be submitted in company with hospital nurses.

The detailed record of maternity-ward work in the Metropolitan and some adjoining Unions (thirty-four in all) deals with 2,935 cases; 1,322, or nearly half of the patients, were primiparæ. In 1,818 cases the infants were illegitimate, including, it must be presumed, those of all, or nearly all, the 991 women who were stated to be domestic servants. The percentage of infants dying during the first two weeks of life is higher than in the general population, but these deaths are fully recognised as being " due to pre-natal and maternal conditions. . . . The general type and character of the parents and the conditions under which they have lived are the dominating factors in determining the death-rate."

* Local Government Board's Forty-second Annual Report, 1913. Part I. Cd. 6980. (Wyman and Sons. Price 1s. 4d.)

